# The life cycle of the Siamese shield leech, *Placobdelloides siamensis* Oka, 1917

**DOI:** 10.1371/journal.pone.0244760

**Published:** 2020-12-30

**Authors:** Krittiya Chiangkul, Poramad Trivalairat, Watchariya Purivirojkul

**Affiliations:** Faculty of Science, Animal Systematics and Ecology Speciality Research Unit, Department of Zoology, Kasetsart University, Chatuchak, Bangkok, Thailand; Waseda University, JAPAN

## Abstract

*Placobdelloides siamensis* is a glossiphoniid leech with a short life cycle. In a laboratory setting, ten mature *P*. *siamensis* left their host (a turtle) after feeding for approximately three days and initiated copulation. The adults spent 3–4 days gestating before depositing eggs (272.8±62.9 eggs/clutch; range: 186–359 eggs/clutch). The eggs then changed from a creamy white to a creamy brownish color before hatching. Hatching occurred after incubation on the parent's ventral surface for 5–7 days. The transparent brood, with a single pair of red eyes, spent a couple of weeks under the venters of their parents. After this period, they left their parents and grew to maturity in 10–15 days; leeches were considered mature when their color was similar to that of their parents and they performed their first copulation. In addition, the mature leeches survived for 163 days on one feeding.

## Introduction

Historically, glossiphoniid leeches have been favored over European medicinal leeches (*Hirudo medicinalis* Linnaeus, 1758) [1] for the study of animal development [2–4]. Glossiphoniid leeches are easily bred in laboratory conditions and exhibit parental care behavior, which is probably *de novo* in this family [5,6]. During their reproductive activities, individuals seize a partner with their anterior portions and the two leeches exchange pseudospermatophores, which appear to release spermatozoa on the surface of their bodies. Then, thin-walled cocoons, which consist of large yolk-filled eggs, are created. The parent usually covers the cocoons under its body for protection and provision of food for the developing larvae [7].

According to Kutschera and Wirtz (2001) [8], the Glossiphoniidae family can be divided into three subfamilies based on cocoon attachments: Glossiphoniinae (i.e. *Glossiphonia complanata* Linnaeus, 1758 [1]) attach cocoons to substrates, Haementeriinae (i.e. *Helobdella stagnalis* Linnaeus, 1758 [1] and *Helobdella triserialis* Blanchard, 1849 [9]) attach cocoons directly to the venter of the parent, and monogeneric Theromyzinae (a unique genus, i.e. *Theromyzon tessulatum* Müller, 1774 [10]) show a mixture of these characters [8,11,12].

Siamese shield leech (*Placobdelloides siamensis* Oka, 1917 [13]) is a common freshwater glossiphoniid leech in the subfamily Glossiphoniinae that is found feeding on various Thai geoemydidae turtle species, such as *Cuora amboinensis* Daudin, 1802 [14], *Cyclemys oldhamii* Gray, 1863 [15], *Hieremys annandalii* Boulenger, 1903 [16], *Heosemys grandis* Gray, 1860 [17], *Malayemys khoratensis* Ihlow et al., 2016 [18], *Malayemys macrocephala* Gray, 1859 [19], *Malayemys subtrijuga* Schlegel & Müller, 1845 [20], and *Siebenrockiella crassicollis* [21–24]. Previous ecological data suggest that *P*. *siamensis* is adaptable to various freshwater conditions; however, the life cycle of this species, and the *Placobdelloides* genera, is still unknown. Therefore, to improve our understanding of the reproductive status of *P*. *siamensis*, the life cycle and the duration of each growth stage of this species were examined in a laboratory.

## Materials and methods

### Specimen collection

**Fig 1:**
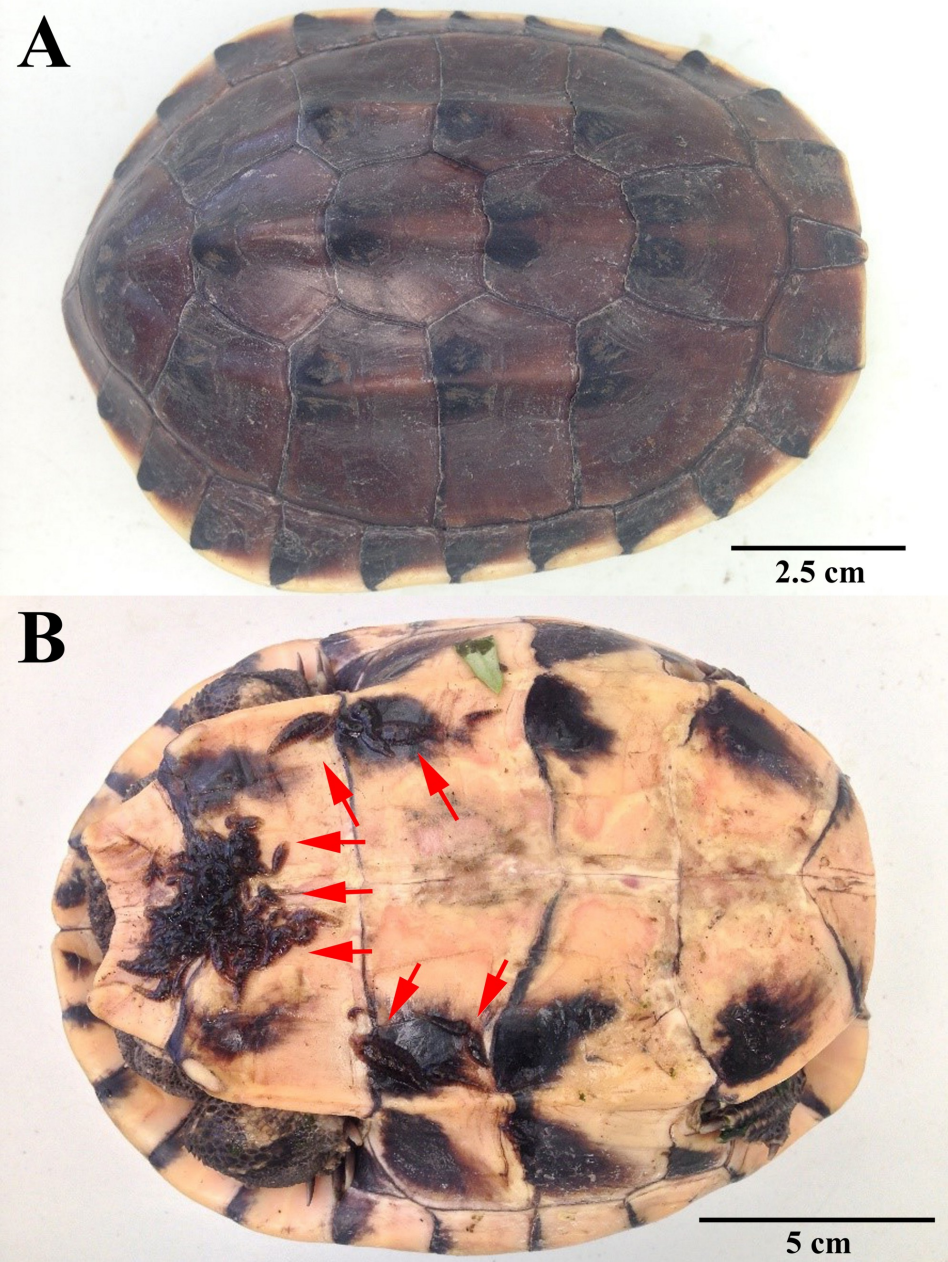
Collected Malayan snail-eating turtle (*Malayemys macrocephala*) with *Placobdelloides siamensis* on the plastron (red arrows).

A mature Malayan snail-eating turtle (*Malayemys macrocephala*) was collected by hand from a pond at Kasetsart University, Bangkok, Thailand (13° 50'53.6" N 100° 33'47.3" E) on 24 July 2019, and delivered to a laboratory in the Department of Zoology, Faculty of Science (Kasetsart University, Bangkok, Thailand) (Fig 1). The turtle has a carapace length of 16.3 cm and a weight of 739 g. The turtle was released at the capture site when the experiment was finished.

All the leeches, totaling 213 individuals, were carefully removed from the external surface of the turtle using forceps and kept separately in a glass container (30x20x20 cm^3^) three-fourths full of water with an oxygen pump at room temperature. A single leech species, *Placobdelloides siamensis*, was identified following Chiangkul et al. (2018) [22]. Ten mature *P*. *siamensis* individuals with an average body length of 30.3±5.9 mm (2.19–4.06 mm, n = 10) were randomly transferred to the turtle container. The observation and recording of water temperature began on 25 July 2019.

### Laboratory preparation

The turtle was maintained at room temperature in a glass container (57x32x33 cm^3^) that was filled one-third of the way with water; a pot was placed above the water level for basking and an oxygen pump was placed in the water. The weight and carapace length of the turtle were recorded both pre- and poststudy, and Mazuri Aquatic Turtle Diet was provided once a day. Once a week, half the water was drained from the turtle container and fresh water was added until it reached the former level; this process was performed once a month for the leech container. Ten *P*. *siamensis* individuals were transferred into the turtle container for blood feeding, and their life cycles were observed. Four periods were recorded: the copulation period (attachment of the posterior sucker to the substrate and body-wiping against the partner's genital pore region), incubation period (from the first egg deposition to the first hatching), parental care period (from the last hatching through the first juveniles leaving their parent), and maturation period (from the last juvenile leaving its parent for its first copulation). The water temperature in both containers was measured every six hours (0000, 0600, 1200, and 1800 h) every day.

### Scanning electron microscopy technique

Some leech specimens were dehydrated in a series of ethanol solutions (with two periods spent in each concentration (70%, 80%, 95%, up to 100%); 1 hour for each concentration) using a critical point dryer (CPD; model HCP-2) to reach a critical dry point. The specimens were then mounted on a stub using carbon tape and sputter-coated with gold particles. All of the coated specimens were examined with a scanning electron microscope.

### Ethic statement

This research was approved by the Institutional Animal Care and Use Committee, Faculty of Science, Kasetsart University, Thailand, under project number ACKU61-SCI-028.

## Results

### Copulation, gestation and deposition

**Fig 2:**
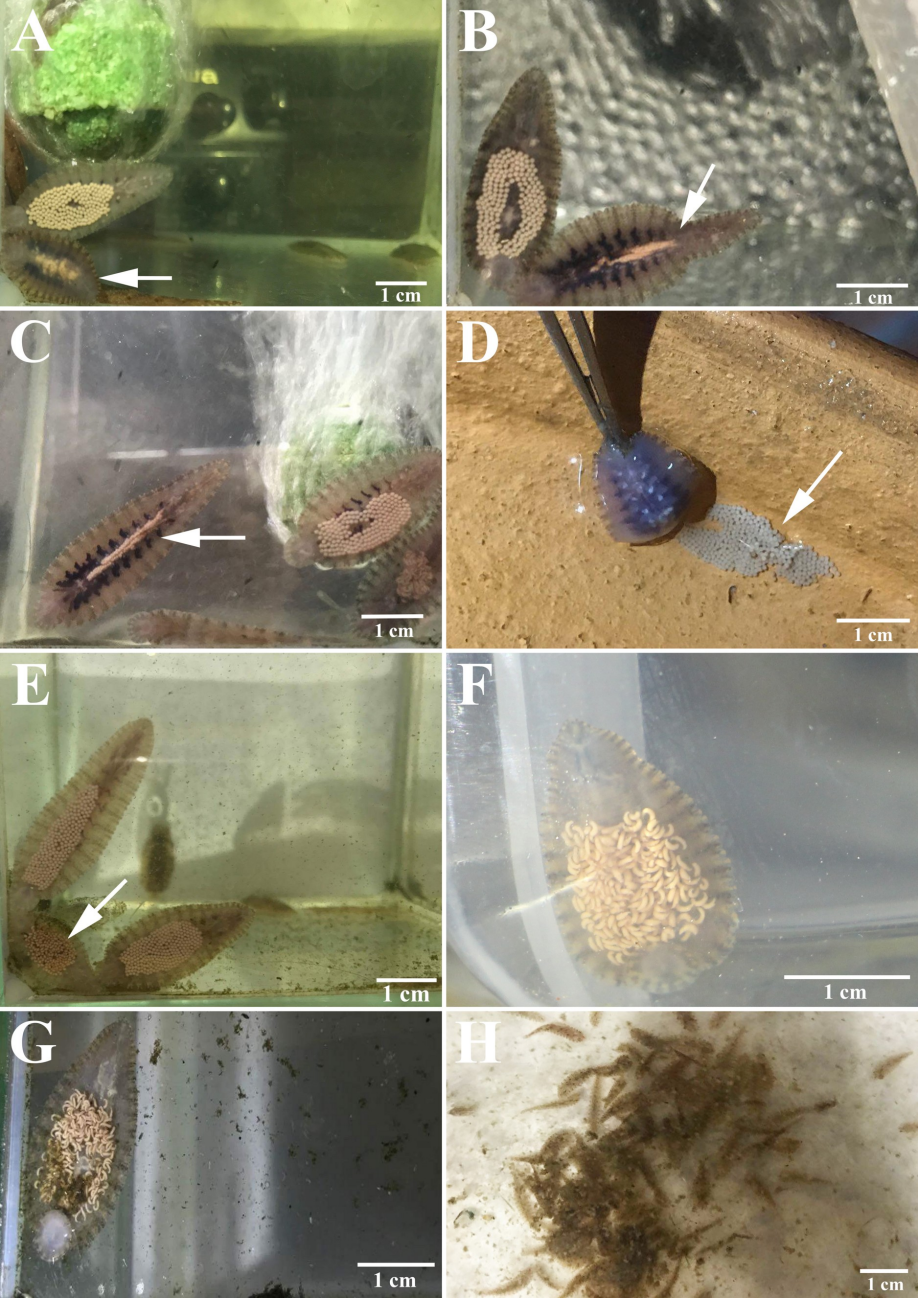
Life cycle of *Placobdelloides siamensis* in the laboratory: gestation period (eggs in ovisacs on the (A) second day (white arrow), (B) third day (white arrow), and (C) fourth day (white arrow) after copulation); (D) a clutch of eggs attached to the substrate (pottery) (white arrow); (E) incubation period (eggs on the sixth day after deposition); (F) parental care period (broods on the (F) second day and (G) seventh day after hatching); and (H) maturation period (juveniles in the third week after hatching).

**Fig 3:**
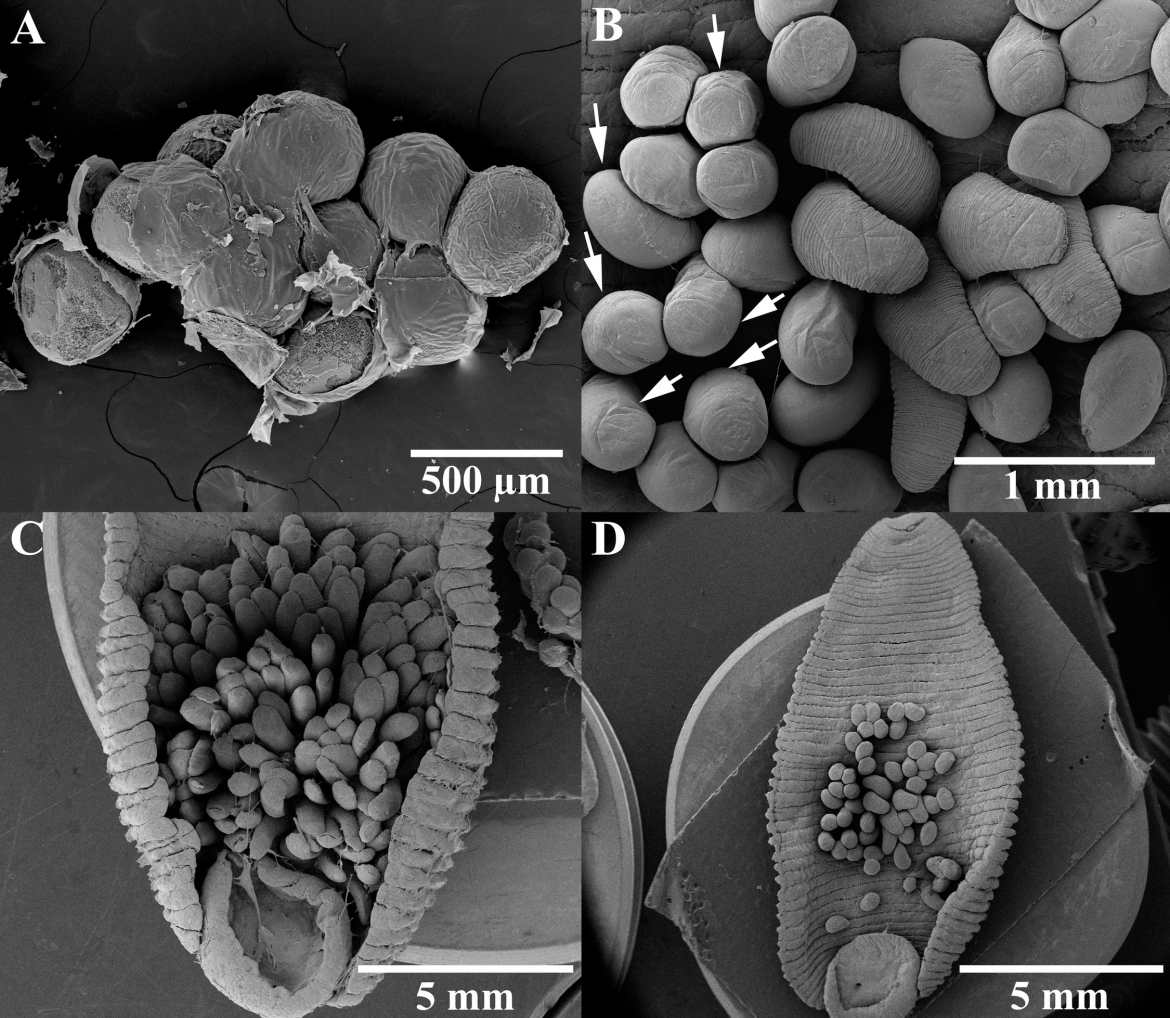
Scanning electron microscopy of *Placobdelloides siamensis*: (A) eggs on the first day after deposition, (B) brood with annulation and nearly hatching eggs (white arrow) on the seventh day after deposition, and brood on the ventral surface of their parents on the (C) second day and (D) seventh day after hatching.

Throughout this observation period (25 July–5 September 2019; 43 days), the average water temperature in the turtle container was 30.1±1.4°C (26.5–34.0°C). On the third day (27 July 2019), after the ten *Placobdelloides siamensis* individuals had fed on the snail-eating turtle (in the turtle container), with blood filling their crop ceca, repeated copulations were observed at the bottom of the container for several hours. On separation, after the last copulation, each *P*. *siamensis* moved toward the substrate near the oxygen pump, where they attached and remained motionless. The gestation of creamy white eggs in both ovisacs occurred one or two days after copulation (Fig 2A–2C). On the third and fourth day of gestation (30–31 July 2019; 6^th^ and 7^th^ day of the observation period), the leeches deposited clutches of creamy white and roundish eggs on the substrates (glass, pottery, or turtle shell) near the oxygen pump and covered them with their bellies (one-third of the way down their bodies, posterior to the venter) (Fig 2A–2D). There were approximately 272.8±62.9 eggs/clutch (range: 186–359 eggs/clutch, n = 10), with an average egg diameter of 452±33 μm (range: 396–514 μm, n = 20; Fig 3A). In addition, when we removed the oxygen pump, the parents started to fan the eggs attached to the substrates with continuous waves of contraction using the flanks of their bodies (S1 Video).

### Incubation and parental care

**Fig 4:**
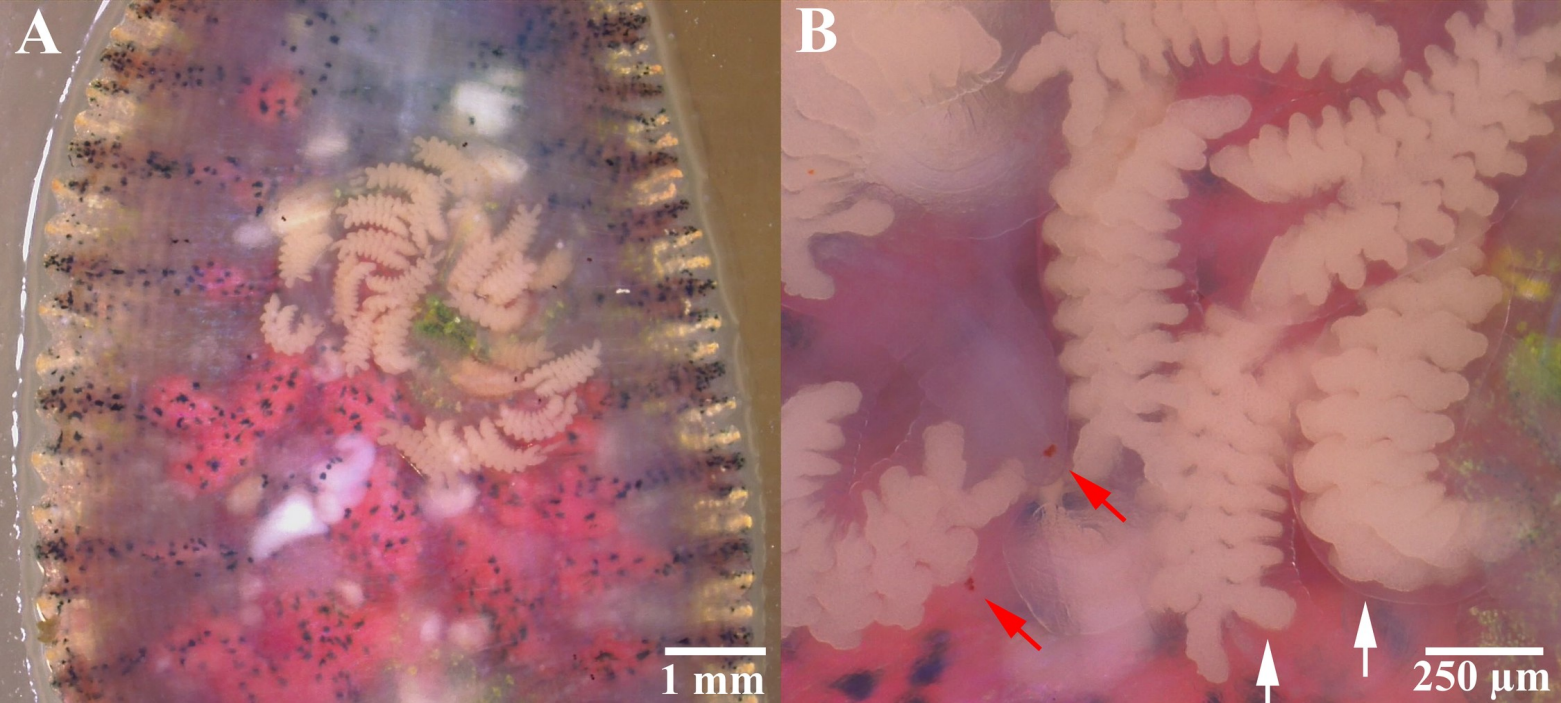
*Placobdelloides siamensis* brood (with transparent bodies, red eyes (red arrows), and yolks (white arrows)) on the ventral surface of their parents on the second day after hatching.

The incubation period lasted 5–7 days (1–7 August 2019; 8^th^ to 14^th^ day). Then, the eggs turned a creamy brownish color, signifying their maturation and readiness to hatch (Figs 2D and 4B). After hatching, the broods spent most of the first week on the ventral surface of their parents (8–14 August 2019; 15^th^ to 21^st^ day); movement began in the second week, but the offspring remained on the ventral surface (15–21 August 2019; 22^nd^ to 28^th^ day, Figs 2F, 2G and 4B–4D). The two-week-old offspring were characterized by transparent bodies, red eyes, creamy white ceca, and average body lengths of 1,130.6±156.3 μm (range: 842.6–1,317.7 μm, n = 10, Fig 4). At this stage, they used the yolk in the ceca as the primary source of nutrients, from which they continued feeding themselves. Moreover, the parents continued to cover their broods at the deposition site without movement or feeding, while the blood in their ceca was continuously depleted, beginning with the anterior ceca.

### Maturation

In the third week (22–25 August 2019; 29^th^ to 32^nd^ day), the broods moved out from under their parents and began feeding on the turtle. At this stage, the offspring are considered "juveniles”. Eventually, in the fourth week after hatching (31 August to 5 September 2019; 38^th^ to 43^rd^ day), when the offspring had coloration similar to that of their parents and an average body length of 10.0±2.1 mm (5.5–13.7 mm, n = 20), they initiated copulation (Fig 2H). In addition, after the juveniles left, the parents had a blood meal and lived without blood in their ceca for a time (approximately 2 months) before death.

The turtle was released at the collection site on the 44^th^ day after collection (6 September 2019) with increased carapace length (17.1 cm) and weight (945 g).

### Starvation

**Fig 5:**
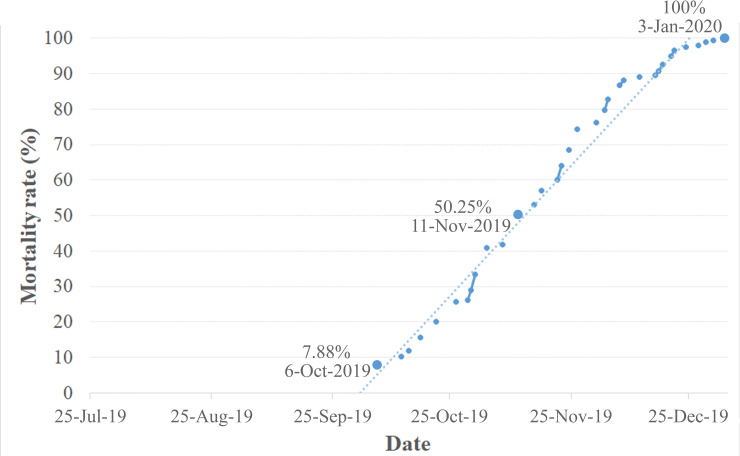
Mortality rate of *Placobdelloides siamensis* after feeding on and separating from *Malayemys macrocephala* from 25 July 2019 through 3 January 2020.

In the leech container, after being separated from the host, some leeches copulated, deposited eggs, and provided parental care as in the turtle container. However, their brood was unable to survive after consuming the yolk within a few days. The death of the mature leeches began in the third month (6 October 2019; 74^th^ day) and reached 53% in the fourth month (15 September 2019; 114^th^ day, Fig 5). Eventually, after 163 days of survival without a host (3 January 2020), the remaining adults died.

Throughout this observation period (25 July 2019–3 January 2020), the mean water temperature in the leech container (starting from 28.9±2.1°C) was as follows: 30.2±1.6°C (28.0–34.0°C) from 25–31 July 2019, 30.1±1.4°C (26.5–33.0°C) from 1–31 August 2019, 30.0±1.4°C from 1–30 September 2019, 28.9±1.0°C (22.0–33.0°C) from 1–31 October 2019, 28.5±1.4°C (26.0–31.0°C) from 1–30 November 2019, 26.9±2.9°C (24.0–31.0°C) from 1–31 December 2019, and 27.1±0.8°C (26.0–28.0°C) from 1–3 January 2020.

## Discussion

This is the first report of the life cycle of *Placobdelloides siamensis* and the *Placobdelloides* genera. This genus is characteristic of the Glossiphoniinae subfamily due to its egg deposition on substrate. In contrast to what was described for this genus in Oka (1917) [13] and Sawyer (1986) [25], *Placobdelloides* are described as brooding the eggs and juveniles on the ventral surface of the parent and the placement of eggs such as *P*. *stellapapillosa* Govedich et al. 2002 [26], and cocoons divides the family Glossiphoniidae into subfamilies. According to this study, we suggests that this genus needs to be re-evaluated and more needs to be done to examine parental care in this family.

**Fig 6:**
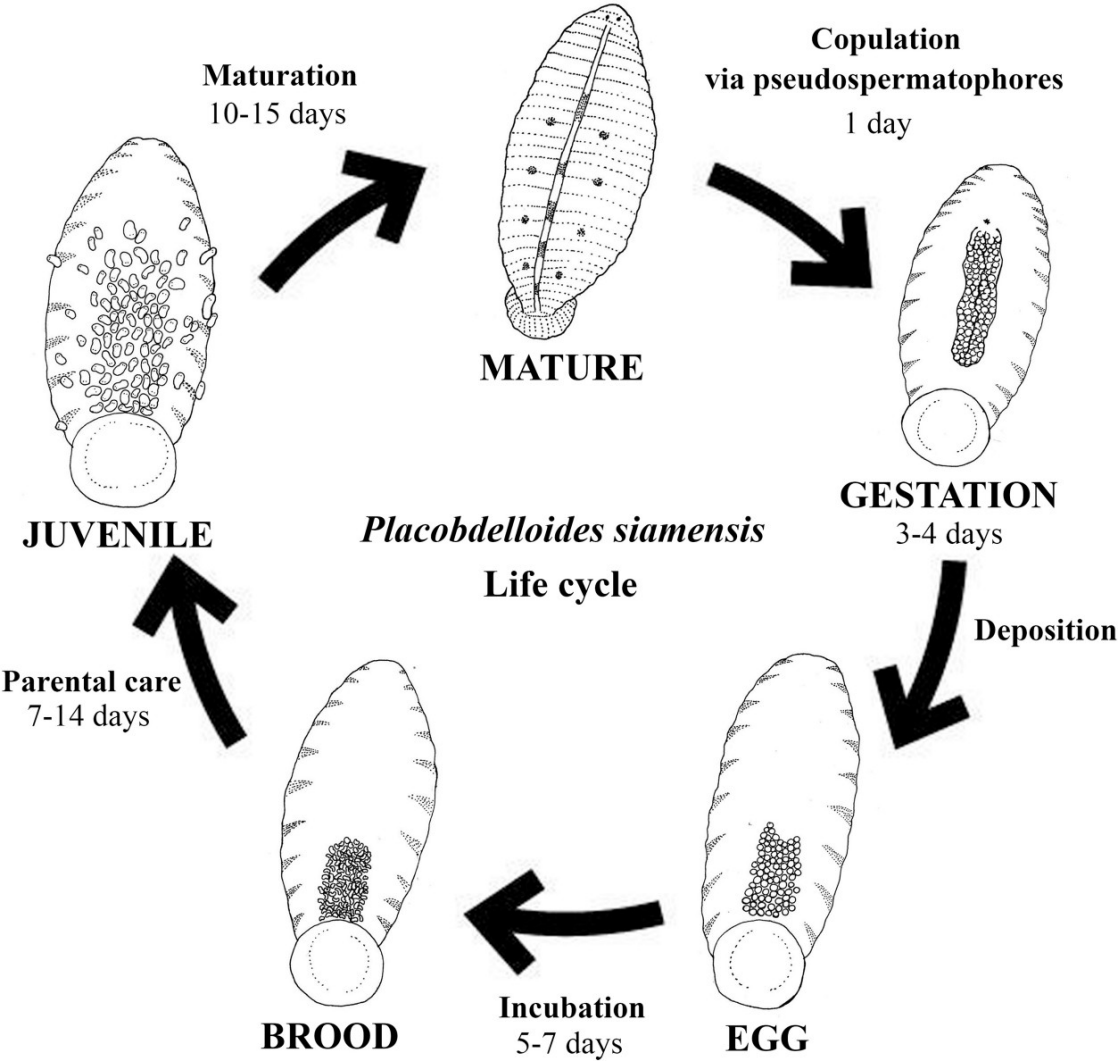
Life cycle of *Placobdelloides siamensis*.

*Placobdelloides siamensis* has a 1-day copulation period, 3- to 4-day gestation period, 5- to 7-day incubation period, 14-day parental care period (from hatching to the juvenile stage), and 10- to 15-day maturation period (from the juvenile stage to the mature adult stage), when individuals are able to reproduce (Fig 6). The life cycle of this species is 33–41 days, and individuals can live semipermanently on the host from copulation through maturation (Table 1).

**Table 1:** Life history data (day) of ten *Placobdelloides siamensis* individuals maintained in a laboratory at 26.5–34.0°C with an oxygen pump and host turtle (*Malayemys macrocephala*).

Periods	Duration (day)
Copulation	1
Gestation	3–4
Incubation	5–7
Parental care	14
Maturation	10–15
Total	33–41

*P*. *siamensis* has a shorter gestation period than *A*. *polypompholyx* Oosthuizen et al., 1988 [27] (30 days), *T*. *tessulatum* Müller, 1774 [10] (6–14 days), and *T*. *rude* Baird, 1869 [28] (5–10 days); a shorter incubation period than *A*. *polypompholyx* (15 days), *H*. *stagnalis* Linnaeus, 1758 [1] (6–12 days), *H*. *robusta* Shankland et al., 1992 [29] (9 days and 13 hours), *T*. *tessulatum* (6–10 days), and *T*. *rude* (5–10 days); shorter parental care period than *G*. *complanata* Linnaeus, 1758 [1] (17–21 days), *H*. *stagnalis* (21–28 days), and *H*. *conifera* Moore, 1993 [30] (60 days); shorter maturation period than *A*. *polypompholyx* (112–140 days) and *H*. *conifera* (35–76 days); and larger clutch size than *A*. *polypompholyx* (30–50 eggs/clutch), *G*. *complanata* (60 eggs/clutch), *H*. *stagnalis* (50 eggs/clutch), *H*. *conifera* (29–192 eggs/clutch), and *P*. *sirikanchanae* (104–115 eggs/clutch; [31]; Table 2) [8,32–35].

**Table 2:** Comparison of glossiphoniid leeches in laboratories, including their host; gestation, incubation, parental care, and maturation periods (days); clutch sizes (eggs/clutch); and water temperature (°C).

Species	Feeding-hosts	Gestation	Incubation	Parental care	Maturation	Clutch size	Temperature	References
Glossiphoniinae								
*Placobdelloides siamensis*	*Malayemys macrocephala*	3–4	5–7	7–14	10–15	173–412	26.5–34.0	This study
*Alboglossiphonia polypompholyx*	*Bulinus truncate*	30	15	7–10	112–140	30–50	18–24	[33]
*Glossiphonia complanata*	Water snail	2–3	4–7	17–21	-	60	Room temperature	[8]
*Placobdelloides siamensis*	-	-	-	-	-	173–412	-	[22]
*Placobdelloides sirikanchanae*	-	-	-	-	-	104–115	-	[31]
Haementeriinae								
*Helobdella stagnalis*	Tubifex worm	2–3	6–12	21–28	-	50	Room temperature	[8]
*Helobdella conifera*	*Helisoma duryi*	-	-	60	35–76	29–192	20	[32]
*Helobdella robusta*	-	-	9 (+13hr)	-	-	-	23	[35]
Theromyzinae								
*Theromyzon tessulatum*	-	6–14	6–10	-	-	383.8	18–20	[34]
*Theromyzon rude*	-	5–10	5–10	-	-	319.3	20	[34]

The short life cycle of this glossiphoniid leech, which includes parental care in the form of covering eggs for protection and fanning them to maintain environmental conditions, and its relatively large clutch size, play a key role in increasing its reproductive rate [8]. Furthermore, the relatively long time (163 days) before starvation following one feeding is another factor contributing to its survival. Thus, the results of this study indicate that *P*. *siamensis* can live for five months, discover a host, and then produce a large number of larvae with only two individuals.

## Supporting information

S1 Video Fanning behavior of Placobdelloides siamensis under low oxygen conditions. (MOV)
